# Correction to ‘Increase of serum pancreatic enzymes during hospitalization for acute heart failure’

**DOI:** 10.1002/ehf2.15245

**Published:** 2025-02-09

Vijver Marlene A. T. Dams Olivier C. ter Maaten Jozine M. Beldhuis Iris E. Damman Kevin Voors Adriaan A. Verdonk Robert C. van Veldhuisen Dirk J. Increase of serum pancreatic enzymes during hospitalization for acute heart failure ESC Heart Fail 2024 11 3656–3661. doi:10.1002/ehf2.14985 39056408 PMC11631317

1. The title of Figure 1 is incorrect. Currently it states: ‘Consort diagram of the study population and sample quantification. Diagram of the final study population following the inclusion and exclusion criteria.’ The second sentence should be removed (this was added as figure legend, but in the title; it is unnecessary).
2. In the legend of Table 2, the comment to the editor/publisher has been incorporated in the text. ‘Simple linear regression shows that patients with a higher NT‐proBNP at baseline exhibit a larger increase in pancreatic enzymes. Subsequently, amylase level at T72 and lipase level atT24 and T72 are also positively correlated with NT‐proBNP. CI, confidence interval; S.E., standard error. Items in bold are highlighting a p‐value <0.05 (i.e. a significant difference). If the editor prefers to display all values in a non‐bold font, we agree to change this.’ The last sentence should be removed.

We apologize for this error.

